# Knee Osteoarthritis Diagnosis: Future and Perspectives

**DOI:** 10.3390/biomedicines13071644

**Published:** 2025-07-04

**Authors:** Henri Favreau, Kirsley Chennen, Sylvain Feruglio, Elise Perennes, Nicolas Anton, Thierry Vandamme, Nadia Jessel, Olivier Poch, Guillaume Conzatti

**Affiliations:** 1 Université de Strasbourg, INSERM, Regenerative Nanomedicine (RNM) UMR 1260, CRBS, 1 Rue Eugène Boeckel, 67000 Strasbourg, France; f_henri3@hotmail.com (H.F.); e.perennes@unistra.fr (E.P.); nanton@unistra.fr (N.A.); jessel@unistra.fr (N.J.); 2 INSERM (French National Institute of Health and Medical Research), UMR 1260, Regenerative Nanomedicine (RNM), FMTS, 67000 Strasbourg, France; 3 Hôpitaux Universitaire de Strasbourg (HUS), Service de Chirurgie Orthopédique et Traumatologique, CHU de Strasbourg, 1 Avenue Molière, 67200 Strasbourg, France; 4 Laboratory of Medical Genetics, INSERM U1112, CRBS, 1 Rue Eugène Boeckel, 67000 Strasbourg, France; kchennen@unistra.fr; 5 Université de Strasbourg, Complex Systems and Translational Bioinformatics (CSTB), ICube Laboratory, CNRS UMR 7357, CRBS, 1 Rue Eugène Boeckel, 67000 Strasbourg, France; olivier.poch@unistra.fr; 6 Sorbonne Université, Laboratoire d’Informatique de Paris 6 (LIP6), CNRS UMR7606, 4 Place Jussieu, CEDEX 05, 75252 Paris, France; sylvain.feruglio@lip6.fr; 7 Université de Strasbourg, Faculté de Chirurgie Dentaire, 67000 Strasbourg, France; 8 Université de Strasbourg, Faculté de Pharmacie, 67401 Illkirch, France

**Keywords:** diagnosis, medical device, osteoarthritis, artificial intelligence

## Abstract

The risk of developing symptomatic knee osteoarthritis (KOA) during a lifetime, i.e., pain, aching, or stiffness in a joint associated with radiographic KOA, was estimated in 2008 to be around 40% in men and 47% in women. The clinical and scientific communities lack an efficient diagnostic method to effectively monitor, evaluate, and predict the evolution of KOA before and during the therapeutic protocol. In this review, we summarize the main methods that are used or seem promising for the diagnosis of osteoarthritis, with a specific focus on non- or low-invasive methods. As standard diagnostic tools, arthroscopy, magnetic resonance imaging (MRI), and X-ray radiography provide spatial and direct visualization of the joint. However, discrepancies between findings and patient feelings often occur, indicating a lack of correlation between current imaging methods and clinical symptoms. Alternative strategies are in development, including the analysis of biochemical markers or acoustic emission recordings. These methods have undergone deep development and propose, with non- or minimally invasive procedures, to obtain data on tissue condition. However, they present some drawbacks, such as possible interference or the lack of direct visualization of the tissue. Other original methods show strong potential in the field of KOA monitoring, such as electrical bioimpedance or near-infrared spectrometry. These methods could permit us to obtain cheap, portable, and non-invasive data on joint tissue health, while they still need strong implementation to be validated. Also, the use of Artificial Intelligence (AI) in the diagnosis seems essential to effectively develop and validate predictive models for KOA evolution, provided that a large and robust database is available. This would offer a powerful tool for researchers and clinicians to improve therapeutic strategies while permitting an anticipated adaptation of the clinical protocols, moving toward reliable and personalized medicine.

## 1. Knee Osteoarthritis as a Public Health Concern

The risk of developing symptomatic Knee Osteoarthritis (KOA) during a lifetime, i.e., pain, aching, or stiffness in a joint associated with radiographic KOA, was estimated in 2008 to be around 40% in men and 47% in women, while this risk in the overweight population (body mass index—BMI > 30) can increase to 60% [1]. Age and obesity are major risk factors for knee osteoarthritis (KOA), but female sex, knee injuries, repetitive joint use, and muscle weakness also contribute to the risk [1,2]. At the same time, after 60 years old, the prevalence of KOA increases by 10% [2]. Furthermore, KOA prevalence is expected to significantly increase as obesity spreads and life expectancy extends. In 2004, the World Health Organization (WHO) estimated that 43 million people worldwide suffered from moderate or severe disabilities due to osteoarthritis (OA) [3]. Consequently, 3.5 million primary total knee arthroplasties (TKA) are predicted each year in the United States of America (USA) [4] by the year 2030. From an economic point of view, the Centers for Disease Control and Prevention (CDC) evaluated the national annual cost at around USD 65.5 billion in 2020, and a rise is expected due to increasing risk factors. Up to now, no curative solution has been found at the cellular or structural level [5]. Various attempts have been made to develop curative treatments—such as microfracture, autologous cell implantation, and scaffold-based tissue engineering—but focused only on the treatment of focal osteochondral defects. In the case of osteoarthritis, apart from joint replacement surgery, medical treatment is focused on hygiene and dietary measures (sport, weight loss), physiotherapy, analgesic/anti-inflammatory drugs, or intra-articular injections, but none of these approaches fully restore tissue health.

The knee is a complex junction made of multiple tissues that must stretch and rotate on multiple axes. Its functionality is made possible through the presence and the complementary action of a liquid (synovial fluid), soft (articular cartilage, meniscus, articular capsule, and synovial membrane), and hard (bones) tissues [6]. The synovial fluid and the cartilage are intended to dissipate energy and limit friction, while the bones maintain a rigid structure of the leg. In more depth, articular cartilage is an avascular connective tissue that is 2–4 mm thick, and it recovers and protects the bones within the articulation joint. It is a complex tissue, composed of water (70–80%), chondrocytes (2–3%), collagen (15–25%), and proteoglycans (5–10%) [7]. Proteoglycans are a combination of glycosaminoglycans (GAGs) and proteins. In the cartilage, aggrecans can mostly be found and play have essential role in shock absorption due to their ability to bind water, thanks to the presence of hyaluronic acid (HA) within their structure, and to maintain a collagen network with strong viscoelastic properties [8]. In addition, to absorb shocks, this tissue has a low-friction coefficient due to the presence of a thin layer of HA on the condyle. This HA is produced by synoviocytes that compose the synovial membrane at the inner surface of the articular capsule and secrete the synovial fluid. In addition, the surrounding synovial fluid, through the presence of HA, has a viscous and dissipative role while maintaining the lubrication of the cartilage surface [9].

During cartilage degradation, various phenomena occur. In OA, there is an imbalance between cartilage degradation and its synthesis, along with a loss of type II collagen and proteoglycans [10,11]. As a consequence, the cartilage tends to lose its robustness and a softening is observed (decrease in elasticity), even in early stages of OA, along with an increase in permeability [12]. This increased permeability—reported to be up to 6.4 times higher than in healthy joints in a rabbit model—is associated with elevated fluid flow, which reduces the cartilage’s ability to resist impact forces [12]. Cracks or fibrous delamination are also associated with cartilage degradation [13]. At the same time, studies have reported a decrease in the viscosity and lubrication of the synovial fluid during KOA, which leads to a loss of its conservative properties. These modifications are associated with a decrease in HA content and molecular weight [9,14,15]. Meanwhile, KOA is usually associated with inflammation, including inflammation and fibrosis of the infrapatellar fat pad [16]. All of these phenomena lead to subchondral bone remodeling, accompanied by hypomineralized tissue, microdamage, and bone cyst formation [17]. Ultimately, KOA results in a highly disabling condition.

From a public health perspective and as suggested by the CDC, early-stage prevention and conservation approaches are recommended in the absence of approved disease-modifying drugs, in order to avoid heavy surgical therapies [18]. Professional Societies’ guidelines have tended to harmonize their recommendations owing to the behavioral conservative strategies and the use of non-steroidal anti-inflammatory drugs (NSAIDs) [19]. However, no universal preservative protocol exists. For example, viscosupplementation can induce a positive response, but it is patient-dependent. Without early markers of OA progression, it is not possible to observe in real time the effectiveness of the proposed treatment. Pain or radiographic evolution are signs of irreversible progression, and clinicians lack easy and responsive tools to better adapt their therapeutic protocols in a reactive way. In addition, there is still an unmet need for the development of curative treatments.

The difficulty in establishing valuable preservative or curative protocols is widely due to the lack of an efficient monitoring method of the joint, and the need for early diagnosis methods is part of the CDC osteoarthritis agenda [18].

## 2. Current Therapeutic Strategies and Limitations

The treatment of cartilage degeneration is mainly symptomatic for pain relief and, once the condition is established, the risk of developing disabling KOA becomes high. As obesity is one of the most significant risk factors for KOA, weight management is a key point on both prevention and treatment of OA, with a combination of diet and exercise, especially when patients suffer from obesity (BMI > 30). For instance, the American College of Rheumatology (ACR) publishes online guidelines that recommend practicing flexibility (stretching), strengthening (leg lifting, pushing…), body awareness (posture, balance…), and aerobic (walking, bicycling…) exercises [20]. Education and self-management of the patient are crucial to limit the disease progression [19].

KOA definition is, however, heterogeneous depending on the diagnosis method (symptomatic, arthroscopic, or radiographic) and is not necessarily associated with pain. The International Cartilage Repair Society (ICRS) scale proposes a macroscopic (arthroscopic) approach to grade the deterioration (from 0 to IV). Grade 0 is considered a normal joint [5]. The first stage of degradation (stage I) is related to a softening of the cartilage (chondromalacia), followed by superficial cracks. Stage II is then defined as cartilage degradation of less than 50% of the cartilage depth, while deeper lesions correspond to grade III, up to the point where the subchondral bone is damaged (grade IV). Grades III and IV are advanced KOA and typically require surgical intervention. TKA, i.e., the replacement of the whole joint with a prosthesis, remains the last stage of treatment. This therapeutic solution is often unsatisfactory, as patients may not fully recover mobility, and residual pain can persist in up to 15% of patients two years after TKA [21]. Furthermore, the limited lifespan of the prosthesis leads to additional heavy surgery.

It is then to explore and develop early therapeutic strategies to avoid advanced KOA. For grades I or II, it is still possible to adopt conservative strategies. Nowadays, patients suffering from stages I or II KOA benefit from NSAIDs and analgesics (e.g., acetaminophen) [14,20]. If not sufficient, opioids can be prescribed, with all the associated risks of addiction. Platelet-rich plasma (PRP) can also be employed without clear evidence of its effectiveness [22]. Alternatively, the injection of HA (viscosupplementation) is intended to increase the viscosity and lubricate the joint, while corticoids are used to reduce inflammation. Their co-administration can further alleviate pain. If this treatment is effective in reducing pain, its effect is limited in time (e.g., 13 to 26 weeks for HA infiltrations [14]), and the positive effects are patient-dependent.

There is no consensus or clear guidelines on which strategy is preferable, with variable results, and the proposed treatments are mainly symptomatic. Usually, radiography is performed to diagnose the KOA and is followed by an attempt to attenuate pain and restore or maintain the joint functionality by infiltration. When further pain appears, the procedure is repeated, based on the symptomology and the clinician’s opinion. At this point, the clinical and scientific communities lack an efficient method of diagnosis to effectively monitor, evaluate, and predict the evolution of KOA before and during the therapeutic protocol.

## 3. Diagnoses of Knee Osteoarthritis

### 3.1. Clinical Practices

**Figure 1:**
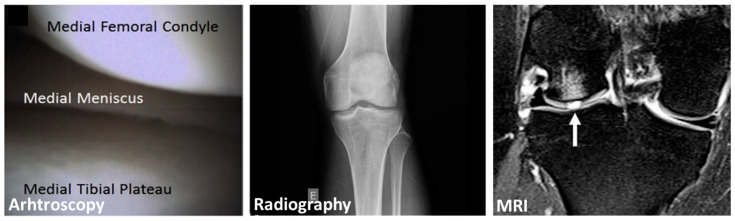
Standard diagnostic methods of KOA. Reproduced with permission from ref. [27] (Arthroscopy), ref. [28] (Radiography), and ref. [29] (MRI). On radiography, “E” is an indicator for “Left”. The arrow points to a subchondral bone marrow oedema-like signal associated with a (grade IV) lateral femoral condyle cartilaginous defect. Reproduced under the Creative Commons License (https://creativecommons.org/licenses/by/4.0/, accessed on 1 March 2025).

Diagnosis of KOA is a hot topic of research due to the difficulty of easily obtaining a clear picture of the joint state. Until now, procedures are based on an arthroscopic, radiographic, or magnetic resonance imaging (MRI) evaluation (Figure 1). Arthroscopy is an invasive method where an endoscope is inserted within the joint to evaluate the cartilage aspect and its stiffness. Although the associated risk is low, arthroscopy should generally be avoided unless required for diagnosing another pathology (e.g., a meniscal lesion), with non-invasive techniques being the preferred option. X-ray radiography has a limiting resolution between 3 and 5 line pairs per millimeter and is one of the standard diagnostic methods, and allows determining the joint space narrowing (JSN) [23]. This technique has drawbacks, as it is limited to bone visualization and only shows the joint space without providing an image of the cartilage or synovial fluid. With a spatial resolution from 13.7 to 540 μm [24], MRI has gained interest as it allows visualizing the whole joint, including cartilage, menisci, and other soft tissues, and does not entail radiation exposure, unlike classical X-ray radiography or computed tomography; however, it still remains difficult to interpret [25]. MRI is considered the most accurate imaging method, but the cost and the difficulty of correlating its findings with clinical signs should be carefully considered [26]. All of these methods primarily detect structural changes, such as JSN, which typically manifest in the later stages of the disease. They show poor correlation with patient-reported symptoms such as pain and stiffness, and lack sensitivity to early cartilage damage or biochemical alterations. Moreover, as they are difficult to implement in preventive routine care, traditional diagnostic approaches are generally used only after clinical symptoms emerge, limiting the potential for early-stage prevention.

KOA diagnosis is usually performed through scoring and grade ranking, depending on the method used. For example, arthroscopic methods rely on Outerbridge, Béguin, and Locker, or more often on the ICRS grading system. Scoring systems possibly allow refining the diagnosis. Radiographic evaluation relies on the Ahlbach or the Kellgreen & Lawrence (KL) scales, but both are based on the JSN evaluation. However, if diagnosis at a critical point is difficult, it allows determining and grading arthritic pathology at a specific point (described above from I to IV).

From this baseline, treatments can be administered to the patient. Despite its crucial importance, their positive effect is nowadays not well evaluated due to the lack of efficient joint monitoring. Furthermore, diagnosis is mainly based on grading systems, and a slight evolution, whether positive or negative, cannot be detected through classical methods. Hence, until now, it is not possible for the medical staff to evaluate and adapt the protocol unless the patient experiences worsening pain or stiffness. Consequently, symptoms or radiographic progression are signals that the treatment is ineffective, but they also indicate tissue degradation, in other words, irreversible KOA progression, with no anticipative possibility. A reliable assessment of the efficiency of infiltrative treatments and real-time monitoring is required to develop new therapeutic protocols, or by adapting standard procedures (personalized medicine), or by developing innovative approaches. Adaptation of the treatment could be undertaken by adapting the strategy, for example, by replacing HA viscosupplementation with an anti-inflammatory treatment, or by adjusting doses and frequency.

However, without a method for kinetically monitoring the state of the tissue, both positively and negatively, any adaptation of therapeutic protocols or personalized medicine would appear to be compromised and remain a hard challenge.

Obviously, the optimal time to perform or adapt treatment administration is before the onset of pain, as pain is a signal of rubbing and tissue deterioration. Decision making would therefore have to be in advance of this, and a relevant signal needs to be envisaged. New biomarkers need to be identified and thoroughly understood to be effectively utilized in such a clinical adaptation. Joint monitoring should be frequent, easy to perform, and non-invasive to minimize patient stress and maintain a favorable benefit–risk balance. A critical challenge is then to obtain reliable tissue information without relying on invasive or complex techniques.

### 3.2. Emerging Diagnostic Methods

To evaluate the progression of KOA, the following tissue markers should be monitored: cartilage softening, appearance of cracks or delamination, decreased viscosity of the synovial fluid, and inflammation status. Despite the difficulty in directly assessing these without invasive observation, indirect methods were implemented.

Methods such as optical coherence tomography (OCT) or streaming potential integral (SPI) arthroscopy permit precise measurements (OCT: lateral resolution 10 µm, depth of penetration: 1.5 mm; SPI: Not applicable) but are invasive [24]. Ultrasound (lateral resolution 30 µm, depth of penetration: 1.65 mm) [24] was reported for joint evaluation due to the ability of collagen and chondrocytes to strongly scatter acoustic signals [30,31]. However, this method still requires the introduction of a probe within the joint and, while it penetrates within the cartilage up to few millimeters, the global observation of a joint would require hundreds or thousands of acquisitions (acquisition window 1 × 4 or 2.8 × 4 mm), limiting the investigation of a specific area [24].

**Figure 2:**
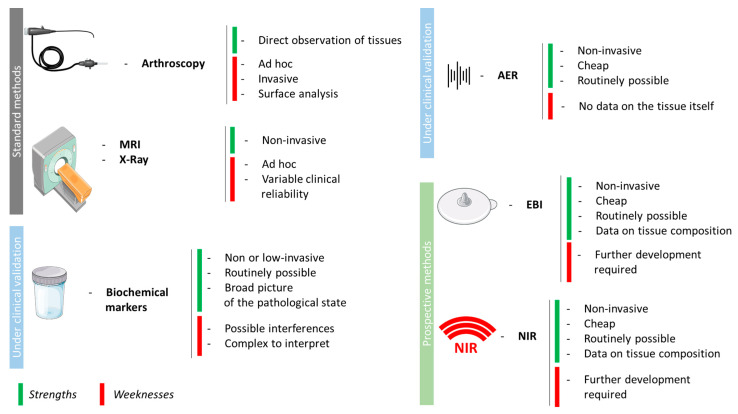
Current under-validation and prospective diagnostic methods. MRI: Magnetic Resonance Imaging, AER: Acoustic Emission Recording, EBI: ElectroBioImpedance, NIR: Near-InfraRed. Combined with material from Servier Medical Art.

Then, other strategies were explored by researchers and medical staff and are described in the sections above (Figure 2).

#### 3.2.1. Biochemical and Physicochemical Markers

Synovial fluid could be used as a biological marker, as changes in its viscosity, pH (a potential early infection marker [32]), ion concentration [33], hyaluronic acid concentration, and chain length [15] characterize the state of KOA evolution. Multi-omics analyses of OA synovial tissues and fluid have shown elevated levels of ornithine, proline, and hydroxyproline—markers of cartilage degradation—alongside increased concentrations of phenylalanine and tyrosine metabolites, and upregulated expression of type I collagen [34]. These findings indicate that patients with knee osteoarthritis (KOA) exhibit active metabolic remodeling, involving simultaneous cartilage degradation, repair, and osteogenesis. This is further supported by elevated levels of fibronectin 1 (FN1), COL1A1, COL3A1, and transforming growth factor β1 (TGF-β1). Other biomarkers such as chemerin, an adipokine, have also been shown to be elevated in pathological knees [35]. In 2021, a systematic review encompassing data from 13,557 patients across 159 articles concluded that the most promising synovial fluid biomarkers for knee osteoarthritis were inflammatory markers such as interleukins (IL-6, IL-8), tumor necrosis factor-α (TNF-α), as well as leptin, matrix metalloproteinases (MMP-1 and MMP-3), tissue inhibitors of metalloproteinases (TIMP-1), and vascular endothelial growth factor (VEGF) [36]. The review also highlighted the lack of consensus and the challenges in achieving robust biomarker validation. Moreover, synovial fluid or tissue analysis requires joint aspiration or biopsy, limiting its practicality for routine clinical use.

Accessible biomarkers were investigated in the field of KOA, and inflammation appears to be a useful indicator to guide the administration of HA or drugs in the joint [37]. Nevertheless, it is necessary to obtain a reliable evaluation, qualitative or quantitative, of a specific biomarker through a rapid analysis. Specific biosensors are complex and usually need to be regenerated. In another diagnosis area, glucose continuous monitoring has overcome this problem by using an electrochemical signal to determine the level of glucose in the blood. Using the same idea, electrochemical sensors were developed for the detection of inflammatory biomarkers such as TNF-α [38,39] or receptor activator of nuclear factor-κB ligand (RANKL) [40]. These markers could be of interest as they are detectable in the circulating system, urine, and saliva, but they lack specificity for KOA.

Other biomarkers present in accessible fluids were investigated for the evaluation of KOA, as presented in Table 1. This table was drawn from a study that analyzed data from clinical cross data stemming from the Osteoarthritis Initiative cohort (biomarker data: n = 600; radiographic and clinical data: n = 4796), [41] and is supported by findings from other studies [42,43,44,45,46] and literature reviews [47]. Among others, we could mention serum N-propeptide of collagen IIA (PIIANP), which relates to cartilage synthesis and whose absence is related to disease progression, or the presence of urinary C-terminal cross-linked telopeptides of collagen type II (CTX-II), which is associated with the degradation of this collagen. Urinary CTX-II has already been identified as a predictive biomarker for 24-month disease progression (i.e., pain worsening and/or JSN loss), along with others such as serum HA [42]. The same study found that a combinatorial model including urinary CTX-II, serum HA, and serum crosslinked N-telopeptide of type I collagen (NTX-I) was the most predictive of OA status. The link between HA and inflamed joints was also reported elsewhere [46].

In addition, IL-6 and C-reactive protein (CRP) have been reported to be biomarkers of synovial inflammation and rapid degradation, but this assertion is not consensual, with contradictory studies [41,48,49]. For example, the correlation of cumulative loading (Physical Activity Scale for the Ederly—PASE—x BMI) and biomarkers such as Cartilage Oligomeric Matrix Protein (COMP), nitrated peptide of type II collagen (Coll-2-1 NO2), with the two-year evolution of KOA (cartilage thickness) is not clear [50]. Many other biomarkers have shown potential, such as matrix metalloproteinase-derived degradation products of type II collagen (C2M), MMPs, sCOMP, micro RNAs (miRNAs); however, standardization and personalization remain to be achieved, particularly in relation to other clinical indicators such as radiographic findings or MRI [47]. Notably, KOA biomarker investigation is a hot topic, and extracellular vesicle surveillance could be incorporated in future studies [51].

Up to now, it is not possible to directly measure these kinds of markers without analytical lab processing, but these markers, if directly correlated with routinely collected data, could further increase the knowledge of KOA evolution and the effect of treatments while helping to develop new monitoring strategies.

#### 3.2.2. Acoustic Emission Recording

Acoustic emission recording (AER, also called vibroarthrography) was reported in 2009 as a method to compare healthy and osteoarthritic knees. It was found that pathological knees produce 6–10 times more acoustic emissions in terms of amplitude, with longer duration (up to 10 times longer) due to higher friction between tissues [52]. Since methods and techniques have widely evolved, and while day-to-day measurements of amplitude and median power frequency were shown to offer low repeatability [53], Nichols et al. published in 2023 a study that employed a wearable acoustic sensing system using a deep noise analysis [54]. The knee acoustic emissions (KAEs) were recorded at the point of care on a cohort of 42 patients with various conditions (healthy, pre-KOA, KOA). The KOA was not radiographic but symptomatic. Based on scripted maneuvers (flexion, sit to stand, and walking), the ability of the device to discriminate KOA and pre-KOA from healthy knees was 94%; this represents a highly promising low-cost option (approx. USD 725) for the diagnosis of KOA, and surely for monitoring its progression over time.

In a direct link to the joint sliding efficiency, AER would allow identification of whether lubrication is required. Up to now, this method has, however, been confined to the discrimination between damaged and non-damaged tissues, whilst the assessment of the joint degradation degree or its precise state still requires further development. Various parameters are also to be considered, such as BMI, muscle mass, and physical activity. For example, a high BMI or age tends to generate higher baseline signals [55], and while some studies mitigate these variations using exclusion criteria, robust models have then to encompass whole-population characteristics.

#### 3.2.3. Electrobioimpedance

Electrobioimpedance (EBI) is nowadays a well-known technology classically used for tissue composition determination. Physicians, but also public people, can now access this technology, which is based on the electric response of the body depending on the administered current frequency, its intensity, and repetition. In short, at low frequencies, the current passes through a conductive (extracellular) environment, while increasing the frequency drives the current through the cells [56]. By varying the frequency, it is then possible to focus on a tissue component, e.g., synovial fluid at low frequencies versus cartilage at higher frequencies. It is worth mentioning that the concept of combining bioelectrical evaluation directly with tissue observation was already investigated through SPI arthroscopy, i.e., passive electrical arthroscopy, with conclusive results demonstrating a relationship between resistance and cartilage health and thickness [24]. Becher et al. successfully correlated SPI values with ICRS and Mankin scores but did not observe a direct correlation with GAG content, likely due to intra-individual and inter-individual variability [57], while other studies have correlated a decrease in streaming potential with a loss of proteoglycans [58]. Compared to SPI, where an electrical signal is passively captured from a mechanically loaded articulation (streaming potential), EBI involves actively applying an electrical current to the tissue and measuring its frequency-dependent electrical resistivity (impedance). EBI measures the variation in impedance with frequency (impedance spectroscopy), providing a more comprehensive profile of tissue composition (water, ions, extracellular matrix).

Recently, a few investigations were made using EBI to evaluate its potential for KOA diagnosis, and some considerations can be reasonably made. First, measurements are usually performed within the frequency range of 10–60 kHz, because it reduces losses due to the skin–electrode interface and allows employing lower current amplitudes to guarantee better safety conditions [59]. Then, when an electric current is applied to a tissue, this latter opposes a force (bioimpedance). It is important to consider that a change in tissue bioimpedance with aging has been reported [60]. Moreover, at 50 kHz, the current passes through both extracellular and intracellular compartments. The reactance (the imaginary part of the impedance) is related to cell number, mass, and integrity, while a decrease in viscosity leads to a decrease in resistance (the real part of the impedance) due to improved ion mobility (Walden relation).

At the same time, this phenomenon has been used to monitor tissue recovery after acute knee injuries (associated with edema) using a wearable setup, moving forward research toward joint monitoring by providing reliable tools and data [61].

**Figure 3:**
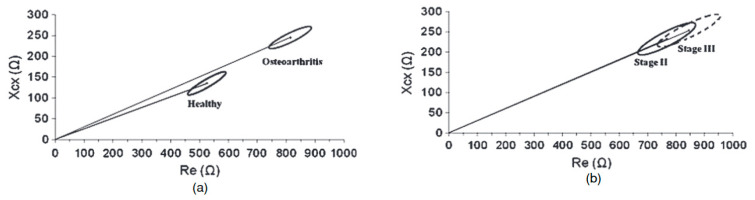
Correlation of BIVA applied to reactance (Xcx) and resistance (Re) for the healthy and osteoarthritic groups (**a**) and for the two osteoarthritic classes II and III of Dejour (**b**). Reproduced with permission of ref. [63].

Using a walking cycle, other researchers validated, in small groups, the relevance of electrical differentiation of healthy joints versus arthritic knees [62]. The study found that subjects with osteoarthritic knees exhibited higher bioimpedance values compared to the control group without OA. In 2010, Neves et al. successfully distinguished OA knees from healthy ones using bioelectrical impedance vector analysis (BIVA) [63]. The BIVA approach enables an assessment of body composition without requiring electrical modeling of tissue components. With a linear correlation between reactance and resistance, they demonstrated that it is conceivable to differentiate radiographic Dejour’s stage II (bones clamping) and stage III (instability and varus), as shown in Figure 3. The authors attributed this to various pathophysiological features of osteoarthritis. In affected knees, factors such as the presence of mineral particles in the synovial fluid, increased synovial fluid volume and pH, elevated leukocyte count, and increased ischemia leading to increased intracellular water uptake all contribute to a rise in resistance (Re) and reactance (Xc) [63]. This study was made with a simple setup, made of a two-electrode acquisition system, and supports the relevance of EBI for joint evolution monitoring, and one can expect a linear progression of the electrical signal during the development of KOA.

**Figure 4:**
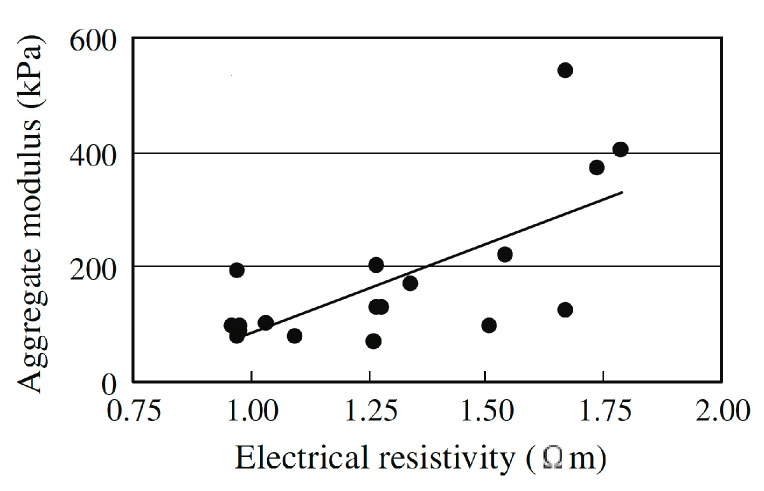
Correlation of the aggregate modulus of degraded pig cartilage with the electrical properties of the tissue. Dots represent experimental data, and the line shows the linear regression. Reproduced with permission from ref. [64].

Variations in electrical response can be attributed to various physiological factors, such as fluid viscosity, but another study has also shown that it can be correlated with mechanical properties [64]. In this study, the authors performed a biopsy of the cartilage of young pigs and applied enzymatic treatments to degrade the collagen and hyaluronate content. Electromechanical analyses revealed a linear relationship between the aggregate modulus (linked to the elastic modulus, i.e., the elasticity) and the resistivity of the condyle (Figure 4). This change in signal response is associated with the increased water content and permeability of degraded cartilage tissue (cartilage lysis), which leads to higher ion mobility and, consequently, a decrease in impedance. This study thus confirms the relevance of electrical bioimpedance for monitoring progression. However, this was the opposite trend compared to the Neves’ study, where healthy tissue exhibited lower resistance [63]; thus, it seems that the differentiation between KOA and healthy knees presented in Figure 4 may not be correlated with the cartilage weakening itself.

There is now strong interest in the electrical characterization of joints, and further studies are needed to develop a comprehensive, reliable, and robust model. Portable systems were manufactured to further use this method outside the lab, with valuable results [61]. Still, these systems require a current supply apparatus, so other studies tried to facilitate the accessibility of this technology by controlling the measuring device using a computer audio board, with conclusive results found to distinguish healthy and injured knees [59].

As for other classical diagnostic methods, the main drawback of EBI is its lack of specificity. As previously mentioned, changes in bioelectrical properties result from variations in hydration (e.g., edema), cartilage weakening, or changes in viscosity. A primary goal would be to identify the tissue source of the signal, for example, its spatialization and differentiation between the fluid, and soft and hard components of the joint (synovial fluid, cartilage and bone, respectively). Electrical Impedance Tomography (EIT) is a method that consists, from multi-channel current recordings, of reconstructing a spatialized representation of the signal. This method was successfully used to monitor lung ventilation efficiency [65]. EIT has not yet been reported for knee joints, but it was shown that it is effective in monitoring other tissues, such as in hand gesture monitoring [66]. Various electrodes are coupled, and a library (Electrical Impedance Tomography and Diffuse Optical Tomography Reconstruction Software—EIDORS) is available to help with spatial modeling. This would be a starting point for developing a new EBI-based monitoring method, providing precious data for clinicians. EIT spatial resolution is determined by the number of electrodes and the considered area, with, for the lung, reported resolutions between 3 and 10 cm [65].

At this stage, it is important to consider both intra- and inter-individual variability, as well as all OA-induced changes in fluid viscosity and volume, tissue composition, cartilage permeability, and the presence of cracks or delamination. In addition to these physiological factors, the experimental setup used (number of electrodes, frequency range, and electrode placement) for data acquisition is crucial. Standardization and a deeper understanding of the pathophysiological influences on the EBI signal are necessary for further development toward robust diagnostic methods.

#### 3.2.4. Near-Infrared Spectrometry

**Figure 5:**
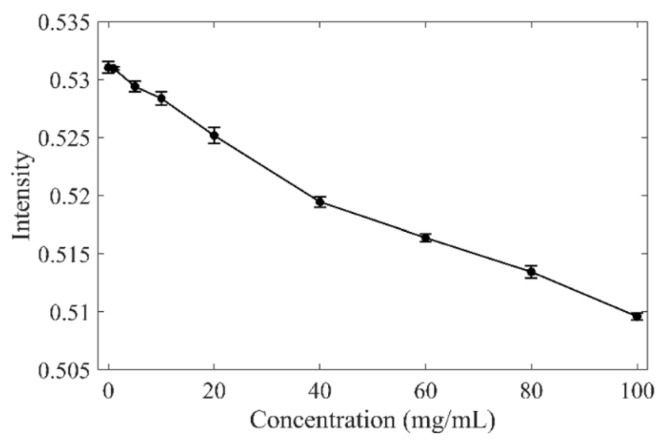
Correlation of absorbance (7930–5930 cm^−1^) with the concentration of hyaluronic acid in water. Reproduced from ref. [68] under the Creative Commons License (https://creativecommons.org/licenses/by/4.0/, accessed on 1 March 2025).

Near-InfraRed (NIR) light is a useful and non-invasive tool for various applications for detection and composition analysis, for example, as a quality control in the pharmaceutical industry or monitoring biological tissues (linked to the blood, especially). One of its unique features is the ability of near-infrared light to penetrate materials relatively deeply (up to several centimeters). This optical spectroscopy can be applied in transmission (through different tissues) or reflection modes. This feature has gained deep interest for upconversion-based in situ drug delivery purposes, and it is also a tool of particular interest for diagnosis. Following the scheme of wearable and external oximeters, which typically utilize two wavelengths (red and NIR) to determine the ratio of oxidized and non-oxidized hemoglobin, the NIR spectrum can also be employed to quantify the composition of the joint. Indeed, the NIR Spectroscopy (NIRS) was proved to be relevant to determine, ex vivo, the cartilage defect in correlation with histologic Mankin [67]. In addition, it has been demonstrated that NIRS allows for the quantification of HA concentration in water, from 0 and 100 mg/mL (Figure 5) [68]. Moreover, normal synovial concentration is around 3 mg/mL. OA is associated with a reduction in HA content, with reported values from 2.26 mg/mL to 1.90 mg/mL, although this concentration is people-dependent [15]. The sensitivity of NIRS would have to be sufficient to finely determine such small variations, and this still remains a scientific challenge. A study conducted in 2023 on human cadaver knees concluded that, by analyzing signals associated with collagen organization (400–600 nm), collagen content (1000–1300 nm), and proteoglycan content (1600–1850 nm), early OA could be detected [69]. It is also worth mentioning that arthroscopic NIRS has been proposed to enhance conventional arthroscopy by offering simultaneous quantitative data on tissue properties, such as cartilage biomechanics or bone mineral density [70]. Despite NIRS’s potential, all of these studies required direct contact between the tissue and the probe, either through ex vivo biopsies or invasive exploration.

NIRS thus appears to be a promising candidate for the detection and monitoring of KOA. Combined with its ability to penetrate, the potential for NIRS to finely assess, within a specific wavelength range, the HA concentration within the joint is a promising insight. For example, as viscosupplementation is one of the standard treatments, it would clearly inform whether an HA injection is required or not. While further development is required, NIRS could potentially become a powerful tool for assessing cartilage composition and health. In addition to EBI, NIRS would also enhance the interpretation of electrical monitoring and help discriminate the underlying causes of impedance changes.

Finally, as an encouraging study, Palukuru et al. used NIRS to efficiently measure the quantity of collagen and chondroitin sulfate on cartilage plugs (articular and nasal) from bovines using multivariate analysis [7]. This study was performed ex vivo, and non-invasive methods still need to be developed.

## 4. Toward Personalized Medicine Through the Implementation of Robust Algorithms

Personalized medicine represents a transformative shift in healthcare, aiming to tailor medical treatments to individual patients based on their unique characteristics. KOA is a condition where personalized approaches are critically needed due to its heterogeneous nature and variable progression [1]. Artificial intelligence (AI) approaches, particularly machine learning (ML) and deep learning (DL), have emerged as promising for risk prediction tasks because they outperform conventional statistical approaches through their capability in finding patterns in different data for diagnosis, predictive modeling, and individualized treatment strategies [71,72].

### 4.1. AI Algorithms in KOA: Diagnostic Accuracy and Clinical Utility

Various AI-driven algorithms have been developed to address different facets of KOA, demonstrating promising accuracy and clinical relevance. These algorithms can process complex datasets in this disease from imaging modalities such as MRI, X-ray radiography, and ultrasonography, detecting subtle pathological features that may not be discernible to human observers. Imaging-based AI models utilizing modalities have already achieved high diagnostic accuracy (Ac), typically ranging from 76 to 90% of accuracy [72,73].

While new techniques have emerged for monitoring KOA, their integration into routine clinical practice remains variable and dependent on multiple factors such as validation level, technical complexity, cost, and regulatory status. Some of these methods are already supported by clinical studies or real-life pilot testing, while others are still under technological optimization. Validating the techniques requires large cohorts and a standardized acquisition apparatus. Table 2 summarizes the main advantages and drawbacks of the emerging methods, as well as their development stage.

Arthroscopy, while showing high specificity (Sp—97%) and accuracy (90%), remains invasive, limiting its widespread use [74]. Owing to X-ray, a study conducted on 59 patients reported a low correspondence between radiographic and arthroscopic observations for early KOA (Ac = 59% for KL grade I) [75]. Efforts were made to develop algorithms able to detect or classify KOA, including early pathology stages. In 2020, it was shown, for instance, that a convolutional neural network (CNN)-based method was as accurate as a fellowship-trained arthroplasty surgeon for radiographic evaluation [76]. Furthermore, radiography processing has shown grading accuracy from 91% to 99% through optimized DL models on 500 manually KL-graded X-ray images [77]. These studies confirm that AI has the potential to efficiently classify radiographs. While this method is cost-efficient and widely accessible, it is expected that, although AI may improve diagnostic standardization, early diagnosis will remain challenging due to the lack of data on cartilage visualization.

Transformer-based architectures, such as iSegFormer, have further enhanced MRI analysis by providing detailed three-dimensional segmentation of cartilage lesions, thus offering critical spatial and temporal insights into tissue degradation that are not typically detectable through conventional methods [78]. Additionally, AI-driven multimodal algorithms combining imaging with clinical and demographic parameters (e.g., age, gender, BMI, inflammatory markers) have further improved predictive accuracy regarding KOA progression, with an Area Under The Curve—AUC = 0.79 [79,80]. In particular, Nelson et al. effectively used machine learning to discriminate KOA phenotypes based on multimodal datasets from the Foundation for the National Institutes of Health (FNIH) cohort study, underscoring their clinical utility in patient stratification and personalized treatment planning [80]. In the end, MRI has the advantage of providing detailed soft tissue imaging, and machine learning processing has shown relatively high accuracy (up to 89% [81]), but remains expensive, while its relevance for early detection still needs to be validated. For example, it reported only 36% accuracy for early OA detection [75]. As for X-ray, AI can help improve MRI diagnostic standardization, but early detection of OA remains a challenge.

Beyond imaging and clinical data, biomarker-based ML models have begun incorporating biochemical and multi-omics data. Ahmed et al. demonstrated the clinical potential of biochemical biomarkers (glycated, oxidized, nitrated proteins, amino acids) assessed in synovial fluid and plasma, significantly enhancing the detection of early KOA, with sensitivity (Se) and specificity of 92% and 90%, respectively [82]. More recently, and taking advantage of the advent of multiple omics approaches (e.g., genomics, epigenomics, transcriptomics, proteomics, and metabolomics) to identify novel biochemical biomarkers and pathways that interplay in the KOA mechanisms, some other initiatives factored in these multi-omics data as main features [83,84,85]. As such, Wang et al. used transcriptomics analysis on peripheral blood samples in combination with several ML models based on biomarkers (B3GALNT1, GRB10, KLF9, and SCRG1) (B3GALNT1: Beta-1,3-N-acetylGALactosaminylTransferase 1; GRB10: Growth factor Receptor-Bound protein 10; KLF9: Kruppel-Like Factor 9; SCRG1: SCrapie-Responsive Gene 1) to predict the early diagnosis of KOA [86]. Biochemical biomarkers offer insights into cartilage metabolism and inflammation, and their analysis represents a promising predictive tool; however, their clinical utility is constrained by systemic variability and the need for an analytic lab.

Novel approaches such as AER and EBI are potentially portable, low-cost, and easy to operate, but their integration into clinical practice will require standardized acquisition protocols and adequate staff training. Notably, AER combined with CNN-based methods has become highly effective in assessing JSN, osteophyte formation, and cartilage damage, with relevant radiographic correlations (AUC = 0.90) [55]. Using the same method yielded an accuracy of 83% for OA diagnosis and 71% for distinguishing between healthy, pre-OA, and OA stages based on ML analysis [54]. The same team validated the AER method using CNN-based models, achieving impressive accuracy—95% for distinguishing healthy individuals from those with OA, and 72% for differentiating pre-OA from healthy cases—highlighting its strong potential for early diagnosis and real-time monitoring of KOA progression [87]. Since manual radiographic scoring is typically used as the reference standard in most reported studies, a bias may exist that partly explains the lower accuracy observed with other methods like AER compared to algorithmically processed radiography [77] (91% to 99% accuracy [77]), especially considering that radiography is an older and well-established diagnostic technique. Nevertheless, the reported values are very promising, and this method could serve as a valuable complement to conventional radiography in the future.

EBI has shown very high accuracy (98%) and AUC (1.00) [88], but remains in early validation stages with limited clinical deployment. The method is inexpensive and potentially suitable for portable setups, but large-scale studies are needed to fully assess its clinical relevance. NIRS shows promise for tissue composition analysis, although its current application is primarily ex vivo, with early-stage development for OA detection using cadaver knees [69]. Its successful use in diagnosing chronic lateral ankle instability suggests potential for reliable, non-invasive diagnostics [89]. Further clinical validation and improved signal interpretation algorithms are needed, but NIRS—along with EBI—could offer valuable insights into tissue composition.

Emerging methods, when combined with appropriate algorithms, have demonstrated robust accuracy that correlates well with classical diagnostic techniques. These approaches are expected to gain further relevance, offering low-cost, user-friendly, and potentially wearable or routinely feasible monitoring solutions with a positive impact on KOA therapeutic protocols. Successful translation into clinical practice will hinge not only on technological performance but also on robust validation across diverse populations, interdisciplinary collaboration, and adequate training of healthcare providers to ensure consistent and reproducible results.

**Table 2:** Summary of the key features of conventional and emerging techniques. D = Discrimination between pathologic or healthy knees, C = classification according to severity grades, Se: Sensitivity, Sp: Specificity, Ac: Accuracy, AUC: Area Under the Curve, ML = Machine Learning, DL = Deep Learning, CT = Computed Tomography. Estimated costs range from $ (inexpensive) to $$$$ (very expensive). * for healthy vs. pre-OA (OARSI 4–5).

Method	Characteristics	D/C	OA/Pre-OA, Reference (Grading Scale)	Algorithm	Reference	Cost	Advantages	Drawbacks	Development Stage
Arthroscopy	Se = 75% Sp = 97% Ac = 90%	D	Preoperative diagnoses	-	[74]	$$	Direct joint visualization; high accuracy; allows simultaneous treatment	Invasive; expensive; surgical risks	Clinically established
Radiography	Se = 40% (KL I) to 50% (KL IV) Sp = 73% to 93% Ac = 59% to 90%	D	Arthroscopic (Outerbridge II–IV)	-	[75]	$$	Widely available; low cost; effective for bone changes and joint space analysis	Poor sensitivity in early OA; no cartilage assessment	Standard first-line method
	Ac = 91% (grade I) to > 99% (grade IV)	C	Radiographic OA (KL ≥ II) and Pre-OA (KL I–II)	DL	[77]				
	Se = 88% Sp = 88% Ac = 88%	D	Radiographic OA (Ahlback grades II–IV)	(CT) ML	[81]				
MRI	Se = 36% (VCS I) to 54% (VCS III) Sp = 79% to 93% Ac = 61% to 85%	D	Arthroscopic OA (Outerbridge II–IV)	-	[75]	$$$$	Non-invasive; detects early soft tissue and cartilage changes	High cost; limited access; contraindications for some patients	Clinically established
	Se = 89% Sp = 88% Ac = 89%	D	Radiographic OA (Ahlbach grades II–IV)	ML	[81]				
Biochemical biomarkers	Se = 92% Sp = 90%	D	Arthroscopic pre-OA (Outerbridge grade I/II + normal radiograph + symptomatic knee)	ML	[82]	$$	Reflect cartilage degradation/synthesis or inflammation; detectable in blood, urine, saliva; used in clinical studies	Often low specificity; influenced by comorbidities and systemic conditions; requires laboratory tests that are invasive or costly	Validated in clinical research, but still limited for routine use.
	AUC = 0.73 using sPllANP + sColl2-1 NO2 + sCOMP + uCTXll AUC = 0.78 when including demographic biomarkers	D	Radiographic OA prediction at 48 months (KL ≥ I)	Multilevel regression	[41]				
AER	Ac = 83–92% for OA detection Ac = 71–72% for healthy, pre-OA, OA classification	C	Pre-OA (pain + KL 0-I) and radiographic OA (KL ≥ II)	ML	[54,87]	$	Non-invasive; portable; low-cost; can discriminate KOA/pre-KOA from healthy knees with 94% accuracy; directly reflects joint friction	Influenced by BMI, age, physical activity; not yet standardized to assess KOA severity	Highly promising, already validated clinically in pilot studies [54,90].
EBI)	Ac = 98% AUC = 1.00	C	Severity Classification g0 (normal) to g4 (scale not defined)	DL	[77,88]	$	Non-invasive; data about hydration, edema, cartilage thickness, or synovial viscosity; potential for spatial representation (EIT); portable setups	Lack of tissue specificity; influenced by multiple parameters, spatial information requires modeling (EIT)	Under active development, with a recent study that reported 98% accuracy for OA detection on a small human cohort [88].
NIRS	Se = 57–89% (45–92% *) Sp = 54–100% (52–85% *) Ac = 69–88% (64–87% *) AUC = 0.77 (0.73 *)	D	Ex vivo, histological evaluation of healthy (OARSI 0–1) vs. OA (OARSI 2–3)	ML	[69]	$$	Non-invasive; deep tissue penetration; possibly inform about composition (HA, collagen, chondroitin content); potential to guide viscosupplementation decisions	Still insufficient sensitivity for small in vivo variations; most data from ex vivo studies; requires robust modeling and signal interpretation	Early-stage development focused on early OA detection using cadaver knees. While in vivo use for OA is still emerging, NIRS has shown promise in diagnosing chronic lateral ankle instability, suggesting potential for reliable, non-invasive diagnostics [89].

### 4.2. Clinical Implications of AI-Driven Personalized Medicine in KOA

Since KOA is a multifaceted chronic joint disease, the application of advanced AI-based algorithms in KOA goes beyond technical capabilities and directly aligns with broader medical objectives, significantly impacting patient care. Enhanced diagnostics through AI can facilitate earlier disease identification, allowing clinicians to initiate therapeutic interventions during pre-radiographic or minimally symptomatic stages. Improved diagnostic accuracy combined with precise phenotyping ensures that treatment approaches can be tailored specifically to patients’ disease profiles, thus maximizing therapeutic effectiveness and minimizing unnecessary interventions.

Integrating multi-modal AI approaches incorporating imaging, clinical data, validated biochemical biomarkers like CTX-II (associated with cartilage degradation) or serum COMP (cartilage turnover), and omics profiling could refine predictions of disease progression and therapeutic responses. This ability could markedly improve clinical decisions regarding interventions such as viscosupplementation (e.g., hyaluronic acid injections), corticosteroid treatments, or other emerging therapeutic strategies. Moreover, incorporating real-time biochemical and biomechanical monitoring through innovative and potentially portable technologies like NIRS and EBI offers dynamic feedback on joint health, enabling timely and individualized adjustments to therapeutic strategies. NIR spectroscopy has shown promise in quantifying HA concentration and assessing cartilage health. Integrating NIR data with AI analysis could provide dynamic, real-time feedback on the biochemical status of the knee joint. Similarly, EBI could monitor changes in cartilage and synovial fluid properties over time, allowing clinicians to detect early signs of therapeutic efficacy or failure, enabling rapid protocol adjustments.

In short, NIRS can assess cartilage composition, AER detects abnormal joint friction patterns, and biomarkers like CTX-II and COMP signal cartilage degradation at the molecular level. EBI offers a portable and low-cost means to monitor joint and fluid shifts. Together, these innovations enable earlier diagnosis, more personalized treatment plans, and potentially delay or prevent invasive interventions while offering new tools for disease-modifying drugs development. The result is better disease management, reduced pain progression, and enhanced long-term mobility and quality of life for patients.

Combined with new sensors, AI-driven wearable technologies further represent transformative potential in KOA care. Continuous real-time data acquisition combined with deep learning could significantly enhance the capability for longitudinal disease tracking, early detection of therapeutic efficacy, and timely intervention adjustments. This continuous, individualized monitoring approach could substantially improve patient adherence and outcomes, aligning perfectly with personalized medicine principles.

### 4.3. Challenges and Future Directions

Despite these promising developments, several challenges must be addressed to realize the full potential of AI-driven personalized medicine in KOA. First, the lack of standardized, large-scale, multi-center datasets hampers the training and validation of reproducible, robust AI models. Establishing comprehensive, multi-modal databases that integrate imaging, biomarker, and patient-reported data is essential. Second, the interpretability of AI models remains a critical barrier to clinical adoption. Black-box algorithms, while powerful, are often met with skepticism by clinicians due to their lack of transparency. Developing explainable AI systems that provide clear rationales is essential for the trust of healthcare providers and patients. Finally, ethical considerations, including data privacy and equitable access to AI technologies, must be addressed.

One of the most transformative applications of AI in KOA is its potential to enable continuous, real-time monitoring of disease progression. Wearable devices collect longitudinal data from patients in their daily lives. When paired with DL algorithms, these wearable sensors not only classify disease severity but also track subtle improvements or deteriorations in joint health. This data, processed by AI, provides insights into the biomechanical and biochemical state of the knee joint, allowing for timely interventions. This capability aligns perfectly with the goals of personalized medicine, where treatments are adjusted dynamically based on the patient’s evolving condition. In this prospect, interdisciplinary collaboration between clinicians, data scientists, and engineers will be essential.

From a regulatory point of view, a 2025 draft document from the Food and Drug Administration (FDA) outlines recommendations for the lifecycle management and regulatory submissions of medical devices incorporating AI [91]. It emphasizes the need to clearly describe the AI system’s inputs and outputs, model architecture, the level and type of staff training required, and the clinical role of the users. The draft also calls for a thorough presentation of validation data, performance metrics, and a comprehensive risk assessment. Outputs should be designed to help users easily determine whether the device is functioning as intended. On the European Union (EU) side, the 2024 AI Act introduces a risk-based regulatory framework for AI systems, similarly requiring clear validation procedures and performance metrics [92]. Together, these USA and EU regulations establish a stringent oversight environment to protect public health by ensuring robust validation of AI-based technologies—particularly critical in high-risk applications like diagnostics. It is now up to the scientific, technical, and business communities to foster trust by delivering robust, well-validated, and clinically reliable AI-driven devices.

## 5. Conclusions

New methods such as NIRS, EBI, and molecular biomarker analysis show promise for improved diagnosis of KOA progression by providing insights into tissue health state. AI has shown its ability to efficiently discriminate healthy and arthritic joints, using both conventional (MRI, clinical data) and biochemical analysis. Combined with innovative methods, it is conceivable to reach models with deep and predictive disease progression evaluation.

At the same time, integrated devices are now able to be developed through the miniaturization of opto-electronic components, and various systems are now available, both at the research and commercial scale (blood pressure evaluation, oximeters, continuous glucose monitoring, etc.). From a medium-term perspective, portable NIRS, EBI, or on-chip biomarker analysis could enable home monitoring with a frequent self-analysis. This would help improve AI algorithms, diminish the impact of missing or bad acquisition, and strengthen the diagnosis, allowing early alerts for both patients and medical staff before irreversible joint degradation occurs. Furthermore, it will be a useful tool to follow in real time the evolution of a therapeutic protocol. Providing direct feedback to patients about how their behavior impacts joint health represents a significant advancement aligned with the CDC’s educational objectives.

Altogether, these innovations may enable earlier clinical intervention, allowing more timely and personalized treatment adjustments. By refining disease detection and monitoring, they hold the potential to improve therapeutic outcomes. Ultimately, such advances could significantly enhance the quality of life for patients suffering from knee osteoarthritis.

However, the 2025 FDA draft and the 2024 EU AI Act both establish strict regulatory frameworks for AI-based medical devices, emphasizing validation, transparency, and risk management. It is now the responsibility of the scientific, technical, and business communities to build trust by delivering well-validated, reliable AI technologies.

## Figures and Tables

**Table 1:** The biomarkers evaluated for their suspected relevance in the progression of KOA evolution, based on ref. [41]. Additional cited references support the pertinence of each specified biomarker.

	Types	Functions in KOA	Measured in	Relevance	References
Cartilage markers					
CTXII	Type II collagen	Type II collagen degradation	Urine	Related to symptomatic and radiologic aggravation	[41,42,43]
Coll2-1 NO2	Type II collagen	Type II collagen degradation	Serum and Urine	Evolution related to symptomatic aggravation	[41,44]
PIIANP	Type II collagen	Cartilage turnover	Serum	Baseline related to radiography, evolution related to symptomatic aggravation	[41]
COMP	Extracellular matrix protein	Cartilage degradation	Serum	Baseline related to radiographic aggravation	[41,45]
Proteases					
HA	Glycosaminoglycans	Maintain high fluid viscosity	Serum	Baseline related to radiographic aggravation	[41,42,46]

## Data Availability

Not applicable.

## List of Abbreviations

**Ac**: Accuracy; **ACR**: American College of Rheumatology; **AER**: Acoustic Emission Recording; **AI**: Artificial Intelligence; **AUC**: Area Under The Curve; **B3GALNT1**: Beta-1,3-N-acetylGALactosaminylTransferase 1; **BIVA**: Bioelectrical Impedance Vector Analysis; **BMI**: Body Mass Index; **C2M**: Matrix metalloproteinases -derived degradation of type II Collagen; **CDC**: Centers for Disease Control and Prevention; **CNN**: Convolutional Neural Network; **Coll-2-1 NO2**: Nitrated peptide of type II Collagen; **COMP**: Cartilage Oligomeric Matrix Protein; **CRP**: C-Reactive Protein; **CT**: Computed Tomography; **CTX-II**: C-Terminal Cross-Linked Telopeptides Of Type II Collagen; **DL**: Deep Learning; **EBI**: Electrobioimpedance; **EIDORS**: Electrical Impedance Tomography And Diffuse Optical Tomography Reconstruction Software; **EIT**: Electrical Impedance Tomography; **EU**: European Union; **FDA**: Food and Drug Administration; **FN1**: FibroNectin 1; **FNIH**: Foundation For The National Institutes Of Health; **GAG**: Glycosaminoglycans; **GRB10**: Growth factor Receptor-Bound protein 10; **HA**: Hyaluronic Acid; **ICRS**: The International Cartilage Repair Society; **IL**: InterLeukin; **JSN**: Joint Space Narrowing; **KAE**: Knee Acoustic Emissions; **KL**: Kellgreen & Lawrence; **KLF9**: Kruppel-Like Factor 9; **(K)OA**: (Knee) OsteoArthritis; **miRNA**: micro RNA; **ML**: Machine Learning; **MMP**: Matrix MetalloProteinase; **MRI**: Magnetic Resonance Imaging; **NIR(S)**: Near-Infrared (Spectroscopy); **NSAID**: Non-Steroidal Anti-Inflammatory Drug; **OCT**: Optical Coherence Tomography; **PASE**: Physical Activity Scale For The Elderly; **PIIANP**: Propeptide Of Collagen IIA; **PRP**: Platelet-Rich Plasma; **RANKL**: Receptor Activator Of Nuclear Factor-κB Ligand; **SCRG1**: SCrapie-Responsive Gene 1; **Se**: Sensitivity; **Sp**: Specificity; **SPI**: Streaming Potential Integral; **TIMP1**: Tissue Inhibitors of MetalloProteinases 1; **TKA**: Total Knee Arthroplasty; **TNF-α**: Tumor Necrosis Factor α; **USA**: United States of America; **VEGF**: Vascular Endothelial Growth Factor; **WHO**: World Health Organization.
